# Assessment of Oral Health-Related Quality of Life (OHRQoL) and Factors Influencing Oral Hygiene Habits in Complete Denture Patients

**DOI:** 10.3390/dj14080515

**Published:** 2026-08-13

**Authors:** Alanoud Alotaibi, Zaina Altahiri, Fajer Aljaser, Ghadeer Alenezi, Dena Kayed Ali, Mirza Rustum Baig

**Affiliations:** 1 General Dentistry, Ministry of Health, Kuwait City 13001, Kuwait; a2181143409@alumni.ku.edu.kw (A.A.); zaina.altahiri@hscd.ku.edu.kw (Z.A.); fajer.aljaser@hscd.ku.edu.kw (F.A.); ghadeer.alenezi@hscd.ku.edu.kw (G.A.); 2 Department of General Dental Practice, College of Dentistry, Kuwait University, P.O. Box 24923, Safat 13110, Kuwait; dali.5@ku.edu.kw; 3 Department of Restorative Sciences, College of Dentistry, Kuwait University, P.O. Box 24923, Safat 13110, Kuwait

**Keywords:** complete dentures, edentulous, oral hygiene habits, oral health, OHRQoL, OHIP

## Abstract

**Objectives:** This study aimed to investigate the sociodemographic factors influencing oral hygiene habits and oral health-related quality of life (OHRQoL) among complete denture wearers at Kuwait University Dental Clinic (KUDC). **Methods:** A cross-sectional observational study was conducted using a self-administered questionnaire and an Oral Health Impact Profile-19 (OHIP-19) instrument to assess oral hygiene habits and OHRQoL in 61 complete denture patients, treated by dental undergraduate students at KUDC, between January 2019 and December 2023. Additionally, a clinical assessment of denture hygiene was performed using the Budtz-Jörgensen denture plaque index for all patients (n = 61). Sociodemographic, general health status, oral hygiene habits, and OHRQoL-related data were collected. **Results:** A total of 37.7% of included participants were aged 51–60 years, with 75.4% being male. Most participants (81.96%) had a high school education or less. Caries was reported as the primary cause of tooth loss (83.6%), and 77.04% recorded irregular dental visits. Only 46.05% of participants demonstrated optimal and/or good denture hygiene. Toothpaste was the most common cleaning method (79.21%), and 70.62% removed their dentures at night. A significant association was found between education level and denture wearing (*p* = 0.035), as well as between gender and the frequency of dental visits (*p* = 0.024). However, no significant relationship was observed between gender and denture hygiene (*p* = 0.52). The OHIP-19 results revealed that poor denture hygiene impacted OHRQoL across multiple domains, including functional limitation, physical pain, psychological discomfort, and social disability. **Conclusions:** Unsatisfactory denture hygiene was prevalent among participants, possibly due to limited awareness or inadequate instructions provided by dental health practitioners. Education level and gender influenced certain aspects of denture care, highlighting the need for targeted oral health education programs and improved patient guidance from dental practitioners. Additionally, poor denture hygiene was associated with reduced OHRQoL, emphasizing the importance of addressing both oral hygiene practices and their psychosocial impacts to improve overall well-being.

## 1. Introduction

Despite advances in dentistry, the number of complete and partial edentulous patients is still substantially large across the world. In 2017, the global prevalence of edentulism was estimated to be 3.3%. Expansion in population size and a higher longevity rate mean that the estimated number of edentulous people has increased to 267 million, offsetting the declining rates in edentulism [1]. Slade, Akinkugbe and Sanders [2] predicted that by 2050, 8.6 million adults in the United States (US) would be edentulous. Complete denture prostheses are used for replacing lost teeth and improving masticatory efficacy and esthetics in addition to maintaining alveolar health status [3]. The literature confirms that the presence of certain medical conditions and the use of medications can have an impact on oral mucosa, the amount of saliva and alveolar bone [4]. Factors associated with the dynamic nature of oral hard and soft tissues related to the use of complete denture prostheses are of importance to the dental practitioner as this knowledge guides accurate diagnosis and the maintenance of oral and general health [5].

Complete denture delivery is not considered the end stage of treatment but the initiation of an extended relationship between the patient and the dental practitioner to maintain optimum health and function. It is of utmost importance that patients return regularly to the dentist for clinical follow-up on denture status evaluation and oral health assessment. To minimize the prevalence of denture stomatitis, the patient is instructed to remove the complete dentures for 6 to 8 h per day [6]. A study investigated the relationship between the awareness of denture cleaning and socioeconomic status, disease, and demographic factors [7] and found that women clean their dentures more often than men [8]. However, in general, a lack of knowledge regarding denture maintenance was often found among edentulous patients [9]. Other studies have reported that complete denture wearers have difficulty in cleaning their dentures due to their limited manual dexterity [10,11], and therefore regular recall appointments are effective in promoting good oral health. It is the responsibility of the dentist to recall and educate their patients periodically [12].

Oral hygiene habits and attitudes may be related to multiple factors, such as education level, gender, socioeconomic status, and age [13]; hence the success of the oral health care of geriatric patients is determined by various factors including but not limited to age-related disabilities and the mental status, socioeconomic background, and educational background of the elderly, besides their medical situation and the drugs being used [14].

In addition to oral hygiene habits, it is essential to evaluate how complete dentures impact patients’ overall quality of life. Oral health-related quality of life (OHRQoL) is a multidimensional concept that reflects how oral conditions affect individuals physically, psychologically, and socially [15]. The Oral Health Impact Profile (OHIP), particularly its shortened versions, OHIP-14 and the OHIP-EDENT (OHIP-19), are valid tools used globally to measure OHRQoL across various populations, including complete denture patients [16]. A recent prospective clinical evaluation confirmed that a patient’s self-reported quality of life metrics do not always correlate directly with their biological presentation, highlighting the need to systematically contrast subjective feedback alongside objective clinical findings [17]. However, there are not enough studies addressing these dimensions within the local region, leaving a significant knowledge gap regarding how complete denture wearers respond to institutional dental care configurations within Kuwait. Assessing OHRQoL using OHIP-19 would provide valuable insights into how sociodemographic factors influence both oral hygiene habits and clinical outcomes in complete denture wearers, treated by undergraduate students at Kuwait University.

The aim of the present cross-sectional observational exploratory study was to investigate the sociodemographic factors that impact oral hygiene habits and oral health-related quality of life among complete denture wearers at Kuwait University Dental Clinic (KUDC). Furthermore, this study aimed to clinically assess dentures to determine denture hygiene using the Budtz-Jörgensen denture plaque index. An additional study objective was to analyze the OHRQoL scores of complete denture patients, in relation to their age and gender, and to investigate the association of oral hygiene condition with the OHRQoL scores, at an exploratory level.

## 2. Materials and Methods

The present cross-sectional observational study was conducted using a self-administered questionnaire and the OHIP-19 instrument. This study involved complete denture patients who attended the Kuwait University Comprehensive Dental Clinic (CDC). Complete denture patients’ records were accessed and retrieved using Titanium dental software (Version 7.4, Titanium Software, Inc., Houston, TX, USA) for the period January 2019 to December 2023.

The study protocol was reviewed and approved by the Ethics Committee of the Health Sciences Center, Kuwait University (Reference no. 487), in accordance with the Helsinki Declaration to conduct this study, as per protocol. An information sheet consisting of the purpose and methodology of this study was provided to all the target study participants. Individuals who agreed to participate were requested to electronically agree to the provided consent form. All individuals were aware that participation is completely voluntary and withdrawal at any stage would not lead to penalties or/and consequences, with the assurance of anonymity.

Participation criteria entailed complete edentulism in one or both jaws and heat-cured polymethyl methacrylate (PMMA) complete removable dental prostheses (CRDPs) worn for at least one year. Exclusion criteria included partial edentulous patients with or without partial dentures, patients wearing complete dentures fabricated from materials other than PMMA, and the patient being unable to provide all the required information, as requested in the questionnaires. The overall health status of the participants was not a contraindication.

**Figure 1:**
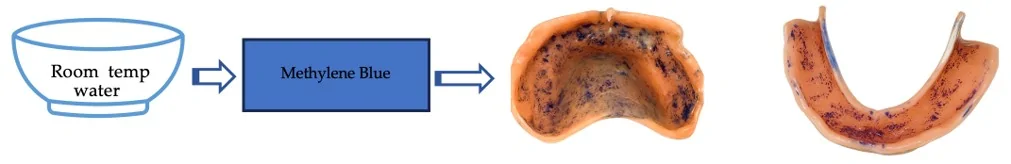
Denture hygiene status assessment.

The questionnaire was piloted on 10 denture wearers who were invited to volunteer in this study, for validation purposes, and inter-examiner agreement, Cohen’s kappa, was found to be 87%. Intraclass correlation coefficients were also tested for intra-examiner reliability, and the values were found to be high, in the range of 90–92%. The purpose of the survey was to provide explanations to the participants, orally and in writing, and written informed consent was obtained. The questionnaire included questions regarding sociodemographic status such as age, gender, and activity, followed by general health status, smoking habits, and the main cause of tooth loss, besides specific questions regarding oral hygiene habits. Additionally, OHIP-19 (OHIP-EDENT) was used to assess the OHRQoL of the participants. This study was carried out on 61 subjects wearing complete dentures. The Budtz-Jörgensen denture plaque index was applied to determine denture hygiene. The assessment criteria were categorized as optimal, good, unsatisfactory, and bad, based on 4 grades of plaque extension on the intaglio (mucosal) surface of the denture: 0 = nonvisible; 1 = less than one-third; 2 = one-third to two-thirds; 3 = more than two-thirds. The maxillary and mandibular complete dentures were soaked in a bowl of room-temperature water for 1 min to remove obvious food debris. The staining of the denture mucosal surface with methylene blue (methylene blue, 0.25% m/v in distilled water; Wako Pure Chemical Industries Ltd, Osaka, Japan) using a syringe, was used to facilitate assessment (Figure 1). The denture hygiene status and habits of denture wearers were analyzed as the primary outcome variables. The secondary variables were oral hygiene habits (with respect to denture hygiene status), sociodemographic characteristics and general health (with respect to oral hygiene habits). The data was discussed and analyzed by a statistical expert. The association between two categorical variables, oral hygiene habits and sociodemographic characteristics, were measured using Fisher’s exact test. For all statistical tests, a 95% confidence level and two-tailed *p*-values were used. Data were analyzed using IBM SPSS Statistics for Windows, Version 25.0 (IBM, Armonk, NY, USA).

## 3. Results

The total number of patients who received complete dentures at KUDC from 2019 to 2023 was 127. Investigators reached out to all the patients, of which 32 could not be reached, 34 refused to participate, and 61 agreed to participate (response rate = 64.21%). The study sample consisted of 46 males (75.40%) and 15 females (24.6%), and 37.7% were aged between 51 and 60 years, and 49.18% were retired. Education-wise, 81.96% of the participants had a high school education or below, and 18.03% had a college degree. The most frequent diseases among the participants were endocrine diseases (64.03%), and 65.57% were smokers. This study found that approximately 72.13% (n  =  44) of the respondents wore complete removable dentures in the upper and lower jaws, and the main reason for tooth loss was reported as caries (83.6%). Furthermore, 22.95% of them visited the dentist regularly, while 77.04% registered infrequent dental visits (Table 1).

**Figure 2:**
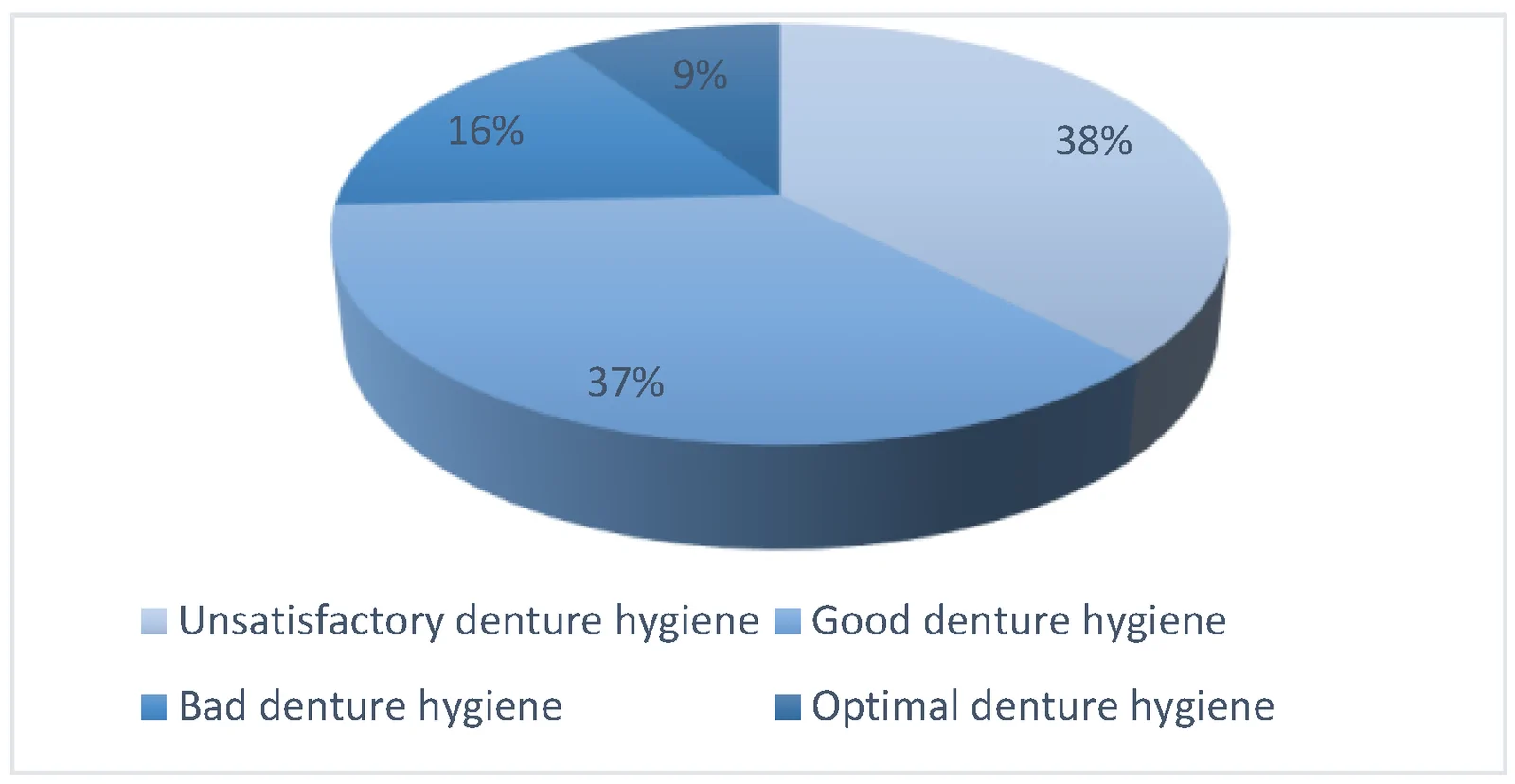
Denture hygiene status.

The study findings showed that 9.41% of the participants had optimal denture hygiene, 36.64% had good denture hygiene, 37.62% had unsatisfactory denture hygiene, and 16.33% had bad denture hygiene (Figure 2).

**Figure 3:**
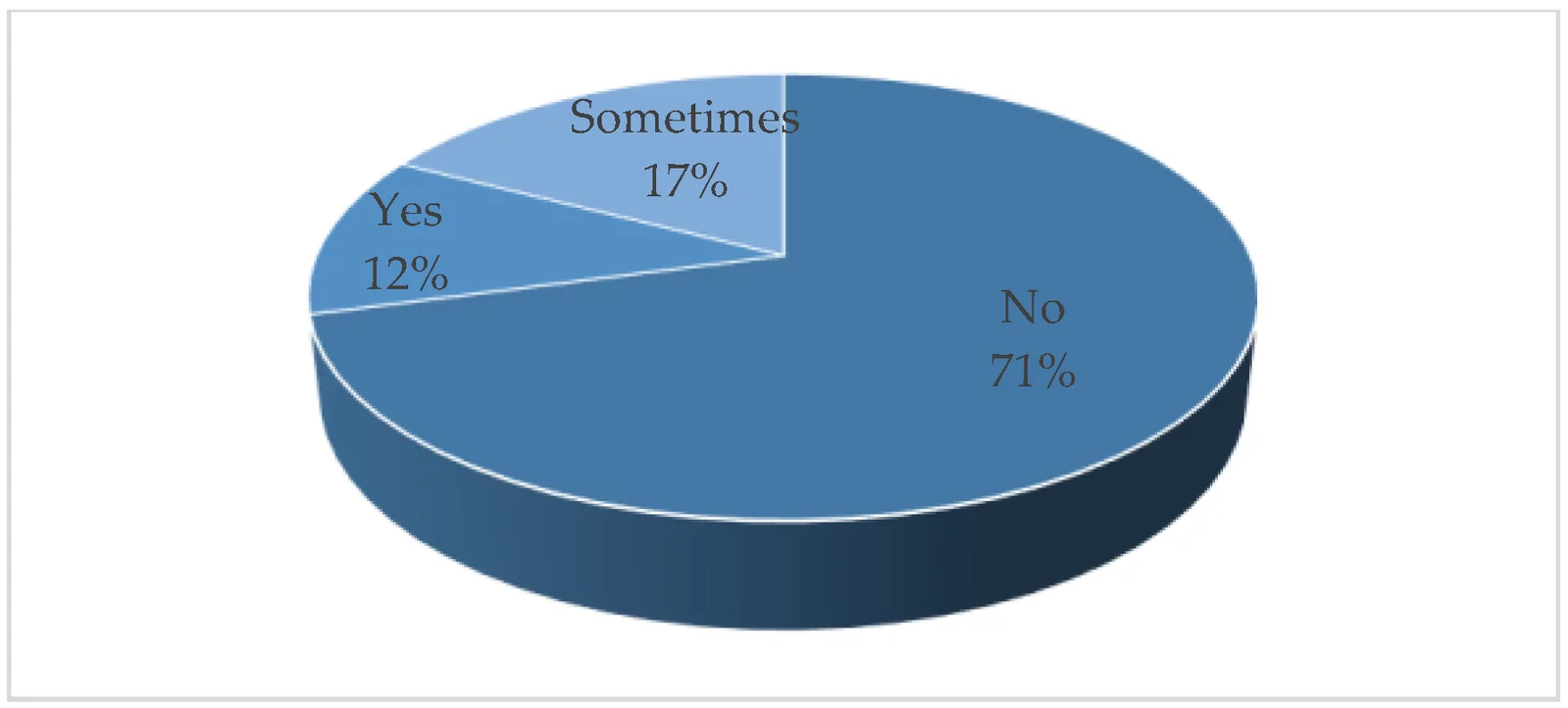
Percentage of patients wearing dentures at night.

**Figure 4:**
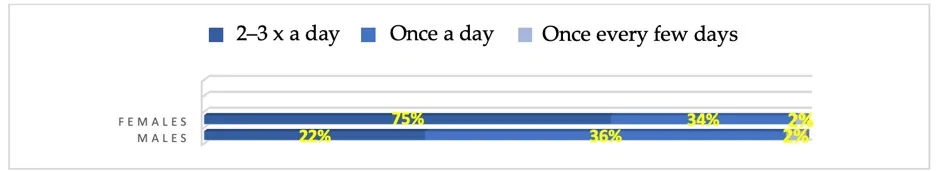
Frequency of denture habits among females and males.

As shown in Figure 3, more than half of the participants (70.62%) removed their dentures at night. Moreover, it was found that most women (75%) cleaned their dentures two or three times a day, and only 34% cleaned them once a day. On the other hand, it was observed that more men (36%) washed their dentures once a day than two to three times a day (22%) (Figure 4).

Education level was significantly associated with denture wearing (OR = 2.43, 95% CI [1.09–5.36], *p* = 0.035) but not with denture hygiene (OR = 1.87, 95% CI [0.93–3.87], *p* = 0.085). Female patients visited the dentist significantly more frequently than male patients (OR = 3.22, 95% CI [1.17–8.24], *p* = 0.024). No significant gender-based difference was observed in relation to denture hygiene (OR = 1.22, 95% CI [0.53–2.74], *p* = 0.52).

As shown in Table 2, the analysis of OHIP-19 subdomains shows that functional limitations were prevalent, with 65.6% of participants reporting chewing difficulty and 60.6% having issues with ill-fitting dentures. Physical pain was also a significant concern, with 44.3% reporting pain in the mouth, 60.7% discomfort while eating, and 60.8% dissatisfaction due to uncomfortable dentures. Psychological discomfort was notable, as 59.1% worried about dental problems. Physical disability was highly reported, with 68.9% avoiding certain foods, 59.2% being unable to eat, and 50.9% experiencing interruptions in eating due to dental problems. Psychological disability was another concern, with 59% feeling upset and 42.6% embarrassed due to dental issues. Social disability affected a smaller proportion of participants: 41% avoided going out, while 44.3% were less tolerant with family and friends, and 41% reported irritability toward others. The handicap domain showed the highest dissatisfaction rates, with 45.9% being unable to enjoy company and 70.5% expressing general dissatisfaction with life.

The OHIP-19 scale ranged from 0 to 76, where higher scores indicated a greater negative impact on OHRQoL. As shown in Table 3, the average OHIP-19 scores varied by age group. Participants aged 41–50 years reported scores of 29.8, followed closely by those under 40 years, at 28.6 (Table 3). Interestingly, individuals aged 51–60 years reported a lower average score of 13.4, indicating better OHRQoL in this group. However, the average score increased again for those aged 61–70 years (31) and decreased slightly for those above 71 years (21.6) (Table 3).

Gender differences were evident in the OHIP-19 scores, with males reporting a higher average score (29.2) compared to females (22.2), indicating that men may experience greater negative impact on their OHRQoL, possibly due to oral health issues (Table 4). This disparity could be attributed to differences in oral hygiene habits, denture maintenance, or other sociodemographic factors.

Denture hygiene influenced the OHIP-19 scores. Patients with optimal denture hygiene had an average score of 28.6, while those with good denture hygiene reported slightly lower scores at an average of 26.27. However, unsatisfactory denture hygiene corresponded to a much higher average score of 43.74, which is comparable to those with bad denture hygiene (43.75). The trend seems to indicate a strong association of denture status with the average OHIP-19 scores (Table 5).

## 4. Discussion

The present study revealed that most complete denture wearers were irregular visitors to the dentist, and more than 80% of denture wearers were found to have caries as the main cause of tooth loss. These findings concurred with Doğan et al. [13] who concluded that one of the common methods of treatment for carious teeth was extraction rather than restorations, in some parts of the world.

The present study investigated the sociodemographic factors that impact the oral hygiene habits of complete denture wearers at Kuwait University Dental Center (KUDC). The population of this study mainly fell in the age group of 51–60 years. This finding was in agreement with other studies [13,18,19], where they concluded that edentulism increased with increasing age. This might be due to the unconservative approach of extracting diseased teeth, especially in older adults believing that tooth loss is a natural part of aging [13,20]. Additionally, most denture wearers in the present study reported smoking habits. Smoking is one of the main risk factors associated with chronic destructive periodontal disease. The typical characteristic of smoking-associated periodontal disease is the destruction of the supporting tissues of the teeth, with the ensuing clinical symptoms of bone loss, attachment loss, pocket formation, and eventually tooth loss [21]. Lamonte et al. and Antonoglou et al. found a strong association between edentulism and cardiovascular disease [22,23]. Eating patterns shift when masticatory performance is compromised. Rough, fibrous foods are avoided or eliminated in favor of foods high in cholesterol and saturated fats, both of which are linked to cardiovascular disease [24]. However, among our study group, endocrine disease was the most prevalent followed by cardiovascular disease. Poor oral health and edentulism can exacerbate endocrine diseases. Caries and periodontal disease are more prevalent among diabetic patients and are the main causes of edentulism [25].

Socioeconomic status has consistently been negatively associated with edentulism. Almost half of denture wearers included in the present study were retired, with 81.96% of all patients having a high school degree or less. Our results revealed a strong connection between the level of education and denture wearers. This is consistent with the findings of Marcus et al. [15], stating that edentulism was negatively related to both education and income level. Those who have attained higher levels of education are shown to have greater financial opportunity and to have a higher priority on dental health [14,26,27]. Education often leads to greater health literacy. It was observed that as the subjects’ educational level increased, so did their level of oral health knowledge [28].

In the current study, the frequency of denture cleaning was higher among females than males. This is consistent with the findings published by Kalyoncuoğlu [29], stating that the frequency of cleaning dentures and using cleansing tablets was significantly higher in females than in males. Gender (female patients) and frequency of dental visits were found to be statistically significant. According to reports from other researchers, females were among the groups more likely to visit dentists or dental clinics [30]. This behavioral trend is further supported by recent cross-sectional data from Jeddah, Saudi Arabia, where female adults demonstrated significantly better oral health practices than males; specifically, 41.5% of female participants attended regular preventive dental check-ups compared to only 35.2% of male participants [31]. These comparable global findings are largely driven by higher esthetic awareness and proactive health-seeking behaviors among women, who prioritize prevention, whereas men frequently delay visits until acute pain occurs. For the same abovementioned reasons, possibly, it could be speculated that the average OHIP scores of the females (22.2) were found to be lower than the males (29.2) in the current study (Table 4).

Our study revealed no significant association between gender (female patients) and denture hygiene, whereas a previous study by Baran et al. found a statistically significant relationship between denture hygiene and the patient gender [32]. No other study in the scientific literature was found to align with the aforementioned finding to the authors’ best knowledge. Although females exhibited better denture hygiene in this study, the difference was not statistically significant. This lack of significance may be attributed to potential limitations, such as the small sample size. Also, the lack of significant gender difference in denture hygiene suggests that general hygiene recommendations are equally effective for both males and females. Individual behaviors, health conditions and adherence to care practices play a more substantial role in denture hygiene rather than gender.

Maintaining the cleanliness of dentures is crucial to prevent malodor, poor esthetics and the accumulation of plaque/calculus, which can have detrimental effects on the mucosa. After an evaluation of denture cleanliness through the fitting surface examination, it revealed that almost half of the patients had optimal or good denture hygiene. This finding is similar to Apratim et al. [33] where half of the patients had good denture hygiene. Bacali et al. [34] also reported 52.4% of the subjects exhibiting good or optimal denture hygiene status. Many patients may not fully understand the importance of proper denture care or it could be failure from the dental practitioner’s side to provide adequate explanations of denture hygiene instructions, leading to inadequate hygiene practices [35].

Of our respondents, more than two-thirds (79.21%) used toothpaste to clean their dentures. Baran et al. [32] revealed that most participants in their study reported cleaning their dentures with a toothbrush and toothpaste and almost half with a toothbrush only. The night wear of complete dentures was found to be significantly associated with the prevalence of denture-related stomatitis [36]. When dentures are worn continuously, it creates a moist environment that promotes bacterial and fungal growth. In the current study, a significant portion of participants reported denture removal overnight. This is similar to a study by Apratim et al. [33], stating that 74.3% of the subjects reported denture removal during the night. To prevent stomatitis, it is recommended to remove dentures at night, clean them thoroughly and maintain good oral hygiene.

The findings of this study underscore the importance of evaluating not only oral hygiene habits but also their impact on oral health-related quality of life (OHRQoL) among complete denture wearers. OHRQoL is a multidimensional concept that reflects how oral health conditions affect individuals physically, psychologically, and socially [37]. The Oral Health Impact Profile (OHIP), particularly the OHIP-19 version used in this study, is a validated tool designed to measure these impacts comprehensively. It captures key dimensions such as functional limitation, physical pain, psychological discomfort, physical disability, psychological disability, social disability, and handicap [17]. The results revealed associations between denture hygiene status and OHIP-19 scores, where participants with poor denture hygiene reported higher impacts across various domains, including chewing difficulty, painful aching, and psychological discomfort. This aligns with previous studies that highlight the correlation between inadequate denture maintenance and reduced OHRQoL [38]. Moreover, demographic factors such as age, gender, and education level were shown to influence OHRQoL outcomes. For instance, the male participants reported higher OHIP scores, indicating greater challenges in maintaining oral health-related quality of life. However, the unexpectedly lower average OHIP scores observed in the 51–60 year age group (13.4) compared to other younger age groups (with recorded average scores of 28–30) could be attributed to the increased awareness, better emotional control, adaptation, and stronger social stability of older adults. These findings emphasize the necessity of targeted interventions to improve denture hygiene practices and enhance patient education [38]. While inadequate or unclear instructions provided by dental professionals may potentially contribute to the poor denture hygiene observed by participants, this interpretation warrants caution as prior patient education was not formally evaluated in this study. Nonetheless, addressing these issues could reduce the functional and psychosocial burdens associated with complete dentures, ultimately improving patients’ overall well-being [39].

The implications of these findings are significant for public health initiatives. Regarding patients’ irregular dental visits and unsatisfactory denture hygiene, there is a clear need for targeted educational programs that emphasize the importance of proper denture hygiene and provide practical guidance on maintaining oral health. Public health campaigns should address common misconceptions and barriers to effective denture care, potentially incorporating community outreach and accessible resources.

Some limitations of the current study must be acknowledged. First, the evaluation was constrained by a relatively small sample size, which may restrict the statistical power of the observed correlations. Second, the study population was exclusively limited to complete denture (CD) patients at Kuwait University (KU), potentially limiting the generalizability of these findings to a broader, more diverse demographic populations. Furthermore, out of 127 eligible patients, only 61 participated. Because non-participants could not be evaluated, those who declined or were unreachable might have differed from our sample, with regard to their oral hygiene habits or OHRQoL, introducing a possible source of selection bias. The imbalance in the number of participants with regard to age, gender, and denture status and the small sample size precluded a statistical evaluation of the differences in the average OHIP scores. Moreover, this study aimed to present preliminary findings. The small number of participants in the optimal denture hygiene category represents an important limitation that may influence the stability and precision of comparisons involving this subgroup. Subsequent studies should be conducted with larger sample sizes to enable us to perform multi-variable analyses to confirm the associations found in this report and to also account for potential confounding. Finally, as this investigation did not incorporate microbiological assessments, future research should integrate microbial testing to provide a more definitive understanding of the specific role microorganisms play in denture hygiene and subsequent oral health outcomes.

Also, future research should also explore the longitudinal effects of improved denture care on OHRQoL and evaluate the efficacy of educational programs tailored to diverse sociodemographic groups and to conduct future studies at the national level by including factors such as perception and attitude.

## 5. Conclusions

In conclusion, this study revealed a high prevalence of suboptimal denture hygiene among complete denture wearers treated at Kuwait University Dental Clinic, which may be attributed to insufficient awareness and/or inadequate instruction and follow-up regarding prosthodontic hygiene practices. Despite most participants removing their dentures at night and relying on toothbrush and toothpaste for cleaning, less than half demonstrated good or optimal denture hygiene. While education level and gender were significantly associated with certain behaviors, such as denture wearing and frequency of dental visits respectively, no significant association was found between these variables and denture hygiene itself. Furthermore, poor denture hygiene was shown to negatively impact oral health-related quality of life (OHRQoL) across several domains, including physical, psychological, and social aspects, as evidenced by the OHIP-19 results. While these conclusions are drawn from a relatively small sample from a single institutional center, the findings carry important practical implications. They underscore the critical need for targeted patient education. Moving forward, dental practitioners must shift from generic advice to actively supporting denture wearers through personalized counseling, explicit maintenance protocols, and structured, long-term follow-up care to ensure optimal denture and oral hygiene, which are essential for overall health and quality of life.

## Figures and Tables

**Table 1:** Sociodemographic characteristics and health status of participants. N = number of patients.

Variable	N = 61	%
**Age**		
<40 years	7	11.47
41–50 years	12	19.80
51–60 years	23	37.70
61–70 years	17	27.86
>70 years	2	3.27
**Gender**		
Male	46	75.40
Female	15	24.6
**Occupation**		
Full-time employed	17	27.84
Part-time employed	5	8.20
Self-employed	0	0.00
Unemployed	9	14.75
Retired	30	49.18
**Education**		
High school or less	50	81.96
College degree	11	18.03
Advanced degree (MSc/PhD)	0	0.00
**Health status**		
Cardiovascular disease	33	54.10
Hepatic disease	10	16.39
Renal disease	13	21.31
Gastrointestinal disease	21	34.42
Endocrine disease	39	64.03
Bone disease	22	36.06
Dermatological disease	8	13.11
Allergies	10	16.39
Other	0	0.00
Smoker	40	65.57
**Type of denture**		
Maxillary	12	19.67
Mandibular	5	8.20
Both	44	72.13
**Tooth loss etiology**		
Caries	51	83.60
Periodontitis	9	14.75
Trauma	1	1.63
Dental visits	14	22.95
Regular	14	22.95
Irregular	47	77.04

**Table 2:** Percentage of subjects responding fairly often, often and very often to each OHIP-19 subdomain.

OHIP-19	%
**Functional Limitation**	
• Chewing difficulty	65.6
• Food entrapment	50.8
• Ill-fitting denture	60.6
**Physical Pain**	
• Painful aching mouth	44.3
• Eating comfort	60.7
• Presence of sore spots	32.8
• Uncomfortable dentures	60.8
**Psychological discomfort**	
• Worry due to dental problems	59.1
• Self-conscious due to dental problems	52.5
**Physical disability**	
• Avoiding some types of food	68.9
• Inability to eat	59.2
• Interruption to eating	50.9
**Psychological disability**	
• Upset due to dental problems	59
• Embarrassed due to dental problems	42.6
**Social disability**	
• Avoid going out	41
• Less tolerant with friends and family	44.3
• Irritable to others	41
**Handicap**	
• Unable to enjoy company	45.9
• Dissatisfaction with life in general	70.5

**Table 3:** Age group and average OHIP-19 scores.

Age Group	Average OHIP-19 Score
<40 years	28.6
41–50 years	29.8
51–60 years	13.4
61–70 years	31
>71 years	21.6

**Table 4:** Gender and average OHIP-19 scores.

Gender	Average OHIP-19 Score
**Male**	29.2
**Female**	22.2

**Table 5:** Denture status and OHIP-19 scores.

Denture Status	Number of Patients	Average OHIP-19 Score
Optimal denture hygiene	5	28.6
Good denture hygiene	21	26.27
Unsatisfactory denture hygiene	23	43.74
Bad denture hygiene	12	43.75

## Data Availability

The data presented in this study are available on request from the corresponding author. The data are not publicly available due to ethical restrictions.
